# Influence of women's decision-making autonomy on antenatal care utilisation and institutional delivery services in Nigeria: evidence from the Nigeria Demographic and Health Survey 2018

**DOI:** 10.1186/s12884-022-04478-5

**Published:** 2022-02-21

**Authors:** Chukwuechefulam Kingsley Imo

**Affiliations:** Department of Sociology, Faculty of the Social Sciences, Adekunle Ajasin University, P. M. B. 001, Akoko-Akungba, Ondo State Nigeria

**Keywords:** Decision-making, Autonomy, Antenatal care, Institutional delivery, Utilisation, Nigeria

## Abstract

**Background:**

In the context of global health priority, understanding the role of power dynamics among women as an important intervention required towards achieving optimum maternal and child health outcomes is crucial. This study examined the influence of women's decision-making autonomy on antenatal care utilisation and institutional delivery services in Nigeria.

**Methods:**

The data for the study were derived from the 2018 Nigeria Demographic and Health Survey and comprised a weighted sample of 20,100 births in the last five years that preceded the survey among married/cohabiting childbearing women. Descriptive and analytical analyses were carried out, including frequency tables and multivariate using the binary logistic regression model.

**Results:**

The study revealed that despite a large number of women initiating antenatal care visits before 12 weeks of pregnancy (75.9%), far fewer numbers had at least eight antenatal care visits (24.2%) and delivered in a health facility (58.2%). It was established that the likelihood of having at least eight antenatal care visits was significantly increased among women who enjoyed decision-making autonomy on their healthcare (aOR: 1.24, CI: 1.02–1.51) and how their earnings are spent (aOR: 2.02, CI: 1.64–2.48). Surprisingly, women’s decision-making autonomy on how their earnings are spent significantly reduced the odds of initiating antenatal care visits early (aOR: 0.75, CI: 0.63–0.89). Some socio-economic and demographic factors were observed to have a positive influence on quality antenatal care utilisation and delivery in a health facility.

**Conclusion:**

In conclusion, women’s decision-making autonomy on their healthcare and how their earnings are spent was significantly found to be protective factors to having eight antenatal care visits during pregnancy. Conversely, women’s autonomy on how their earnings are spent significantly hindered their initiation of early antenatal care visits. There is a need for more pragmatic efforts through enlightenment and empowerment programmes of women to achieve universal access to quality maternal healthcare services in Nigeria.

## Introduction

Generally, pregnancy and childbirth are significant events in a woman’s life and her family even though they represent a period of heightened vulnerability [1]. Proper care during pregnancy and delivery, apart from being a major indicator in measuring the level of healthcare performance, delivery system and developmental indices, it is important for the well-being of both the mother and child’s health. Globally, every day in 2017, preventable causes related to pregnancy and childbirth lead to the deaths of about 295,000 women, with the vast majority of these deaths (94%) occurring in low-resource settings [2]. Between 2000 and 2017, the number of maternal deaths per 100,000 live births dropped by about 38% worldwide and remained unacceptably high in Africa [2, 3].

No doubt, quality antenatal care (ANC) services and delivery in a health facility are important interventions required during pregnancy and childbirth to achieve optimum maternal and child health. These are parts of the key strategies recommended as unique opportunities for the survival and well-being of both mother and child [4–6]. Lack of proper maternal healthcare during pregnancy and childbirth is widely acknowledged to be a major contributor to maternal and child mortality through pregnancy-related complications [7, 8]. The World Health Organisation’s (WHO) guidelines of the four-visit focused ANC (FANC) model which was developed in the 1990s, was probably observed to be associated with more perinatal deaths [9]. This led to the revision of the guidelines stressing that the recommended minimum number of ANC visits from four to eight contacts throughout the pregnancy with the first visit occurring within 12 weeks (first trimester) of gestation [10]. In sub-Saharan Africa, which Nigeria is an integral part of, improving maternal and newborn health remains a major challenge, hence leading to preventable unacceptably high levels of maternal and child deaths [8, 11]. This plausibly could be attributed to power dynamics in the family, especially women decision-making autonomy relating to the utilisation of healthcare services.

Women decision-making autonomy defined as the extent of their independence on finances, matters pertaining to their health and that of the households, and freedom to visit outside the home without having to obtain permission, is considered an important intervention for quality maternal healthcare utilisation [12]. Although, some barriers to the utilisation of maternal health services from demographic and economic backgrounds [13, 14]; socio-cultural and behavioural perspectives [15–17] have been explored. This is specifically in a patriarchal society where women lack autonomy because of financial dependence and cultural norms [18] and men holding primary power has implications for maternal and child health, especially during pregnancy and childbirth [19]. Despite previous research on women’s decision-making power and maternal healthcare utilization in Nigeria [12, 17, 21–31], there have been no studies with a special focus on the timing of first ANC visit, number of ANC visits and institutional delivery services among married/cohabiting childbearing women. Although, there has been continued progress in ensuring universal access to healthcare services, in Nigeria, out of the 57% of women who had at least four ANC visits for their most birth, only 39% delivered in a health facility [32].

This study, therefore, extends the previous studies by investigating the influence of women's decision-making autonomy on antenatal care utilisation and institutional delivery services among married/cohabiting childbearing women in Nigeria. Using the latest Nigeria Demographic and Health Survey (NDHS) data with a special focus on the revised WHO’s recommended minimum number of at least eight ANC visits throughout the pregnancy, mark a departure of this study in terms of its national coverage from most of the previous studies on maternal health services utilization in Nigeria. The outcome of this recent scenario among married/cohabiting childbearing women is expected to provide up-to-date information, relevant policy and programmatic recommendations towards achieving sustainable development goals (SDGs).

## Methods

### Data source and design

The data for this study were obtained from the individual recode data file of the 2018 Nigeria Demographic and Health Survey (NDHS). The survey is a cross-sectional study and data were generated from 41,821 women aged 15–49 and 13,311 men aged 15–59. A detailed report of the data collection methods and procedures for 2018 NDHS has been published elsewhere [32]. The analyses for this study covered a weighted sample of 20,100 births in the last five years that preceded the survey among childbearing women (15–49 years) who were ever-married/living with partners. This is to improve the representativeness of the data from the group of women interviewed in the survey (i.e. 2013–2018).

## Variables measurements

### Outcome/dependent variables

The outcome/dependent variables for this study which was based on empirical evidence were three indicators of maternal healthcare services utilisation during pregnancy and childbirth. These include; 1) timing of first ANC, 2) the number of ANC visits and 3) facility delivery services. Information on these outcome variables was generated from the 2018 NDHS and re-categorised from their original frequency ranges in the dataset. For instance, the women were asked about their first ANC visits during pregnancy, and the frequency ranged from ‘0’ to ‘10’ months. Based on 2016 WHO’s guidelines that first ANC should occur before 12 weeks of gestation (first trimester), timing of the first ANC visit during pregnancy was categorised as adequate (< 4 months) coded ‘1’ and inadequate (4 months and above) coded ‘0’. The second outcome variable, the number of times (visits) a pregnant woman received ANC from a healthcare provider including traditional birth attendant or ANC from a private or public health facility ranged from '0' to '20' visits and was dichotomised as quality (8 +) coded ‘1’ and inadequate (< 8) coded ‘0’. The WHO’s up-to-date recommendation that pregnant women should have at least eight ANC visits is to maximize the benefits of ANC services and is probably associated with greater maternal satisfaction than fewer ANC visits [10]. Thus, those with any associated risk would be required to make more frequent visits to receive quality medical attention [10, 33]. The third outcome variable which is facility delivery/place of delivery is also a dichotomous variable taking the value of '1' if the woman delivered in a health facility (delivery in public or private hospitals or health centres) and '0' if she delivered at home or any other place outside of health facility. There was no distinction made about skilled or non-skilled attendance at delivery because it is assumed that those who delivered in a health facility had skilled attendance.

### Explanatory variables

The main explanatory variables were women’s decision-making measures including the following four subjects: 1) person who usually decides on respondent's healthcare, 2) the person who usually decides on large household purchases, 3) person who usually decides how to spend respondent’s earnings, and 4) the person who usually decides on visits to family or relatives. Therefore, the possible answers were respondent alone, respondent and husband/partner jointly, husband/partner alone, someone else and others. The categories were collapsed into three including ‘alone’ which represents decision-making autonomy, jointly and by combining the last three categories into one (husband/partner alone and others). The selection of women’s decision-making variables was guided by the conceptualisation of the power of a woman to have decision-making authority, including the power to solve problems that can be creative and enabling, especially as it influences maternal healthcare services utilisation [34, 35].

The co-variables likely to influence ANC and institutional delivery services utilisation among women included in the analysis were their current age, educational attainment, work status, wealth quintile, place of residence and region. To make interpretation simpler and more meaningful, some variables were regrouped from their original categories in the datasets. For instance, the current age of the women: 15–24/25–34/35 + years; Educational attainment: no education/ primary/secondary or tertiary; and wealth quintile: lowest/middle/highest. The selection of the co-variables was based on empirical evidence on how they influence access to healthcare services and other aspects of life [12, 21–23, 27, 36–38].

### Statistical analysis

Two levels of analyses (univariate and multivariate) were carried out in this study. At the univariate level, descriptive statistics related to the characteristics of the study population, utilisation of ANC services and place of delivery were generated through frequency. At the multivariate level, unadjusted binary logistic regression analysis was adopted to measure the bivariate relationship between the outcome variables and each of the main explanatory variables, as well as selected co-variables. The adjusted logistic regression analyses were presented and two logistic regression models (Models 1 and 2) were fitted to examine the relationship between the outcome variables and main explanatory variables. Model 1 adjusted for the main explanatory variables to measure the interaction effects of the four main explanatory variables on the outcome variables. Model 2 presented the adjusted logistic regression analysis for the main explanatory variables and selected co-variables. The essence of the two models was to examine the simultaneous interaction effects of the main explanatory variables on the outcome variables in Model 1; and the interaction effects of the main explanatory variables and selected co-variables on the outcome variables in Model 2. Measures of association between the outcome variable and explanatory variables were expressed as OR with 95% confidence intervals (CIs). A variable with OR greater than 1.00 implied that the variable increases the likelihood of the outcome, while it is the opposite when the OR is less than 1.00. All the analyses were conducted using Stata software (version 14). Svy command in Stata was used to adjust for the complex survey design of the Demographic and Health Survey (DHS) data. Besides, the dataset was carefully checked for missing values which were excluded from the analyses and weighted with the appropriate sampling weights as per the DHS sampling scheme before the analyses.

## Results

### Percentage distribution of the variables of the study population sample

Table 1 showed that the largest proportion of the women belonged to those aged 25–34 years, women with no formal education, women who were employed, those found in the lowest wealth quintile households, women residing in rural areas and those living in the North-west geopolitical region.

**Table 1:** Distribution of the sample characteristics, NDHS 2018

	Number (%)		Number(%)
Variables	*N* = 20,100	Variables	*N* = 20,100
**Age (years)**		**Decision on respondent's healthcare**	
15 – 24	4,790(23.8)	Husband/partner alone and other	11,971(59.6)
25 – 34	9,624(47.9)	Respondent and partner	6,382(31.8)
35 +	5,686(28.3)	Respondent alone	1,747(8.7)
**Educational attainment**		**Decision on large household purchase**	
No education	9,206(45.8)	Husband/partner alone and other	12,580(62.6)
Primary	3,075(15.3)	Respondent and partner	6,579(32.7)
Secondary or higher	7,819(38.9)	Respondent alone	941(4.7)
**Employment status**		**Decision on how to spend respondent's earnings**	
Not working	6,496(32.3)	Husband/partner alone and other	1,110(9.3)
currently working	13,604(67.7)	Respondent and partner	2,416(20.3)
**Wealth quintile**		Respondent alone	8,398(70.4)
Lowest	9,362(46.6)	**Decision on visits to family or relatives**	
Middle	4,149(20.6)	Husband/partner alone and other	8,821(43.9)
Highest	6,589(32.8)	Respondent and partner	8,826(43.9)
**Place of residence**		Respondent alone	2,453(12.2)
Urban	6,918(34.4)	**Timing of first ANC visit**	
Rural	13,182(65.6)	< 4 months	11,393(75.9)
**Region**		4 months and above	3,628(24.2)
North-central	3,617(18.0)	**Number of ANC visit**	
North-east	4,241(21.1)	< 8 times	11,393(75.8)
North-west	6,136(30.5)	8 times and above	3,628(24.2)
South-east	2,083(10.4)	**Place of delivery**	
South-south	1,755(8.7)	Home	11,691(58.2)
South-west	2,268(11.3)	Health facility	8,409(41.8)

Overall, the results further showed that 59.6% and 62.6% of the decisions on the women’s healthcare and large household purchases respectively, were made by their husbands/partners. On the other hand, a large proportion of the women admitted having decision-making autonomy on how to spend their earnings. About 44% of the decisions on women’s visits to family and relatives involved their husbands/partners. Concerning ANC utilisation and institutional delivery services, 75.9% of the women initiated their first ANC visit before the end of 12 weeks of pregnancy; 24.2% at least eight ANC visits during pregnancy and 58.2% delivered at home during their last pregnancies in the five years before the survey.

### Antenatal care visits and institutional delivery by women’s characteristics

Table 2 presents the results of the proportions of women who initiated their first ANC visit before 12 weeks of pregnancy, who had at least eight ANC visits and delivered in a health facility by socio-demographic and household decision-making characteristics.

**Table 2:** Proportion of women who had first ANC visit before 12 weeks of pregnancy, adequate ANC visits and delivered in a health facility by explanatory variables, NDHS 2018

Variable/category	N	Timing of first ANC visit (%)	Number of ANC visit (%)	Facility delivery (%)
**Age**				
15 – 24	4,790	23.7	12.6	34.1
25 – 34	9,624	24.6	20.5	44.4
35 +	5,686	23.7	21.3	44.0
**Educational attainment**				
No education	9,206	15.9	4.2	15.2
Primary	3,075	23.3	18.6	44.5
Secondary or higher	7,819	30.4	36.1	72.1
**Employment status**				
Not working	6,496	20.3	10.1	29.6
currently working	13,604	25.7	23.0	47.7
**Wealth quintile**				
Lowest	9,362	19.3	6.0	18.5
Middle	4,149	22.8	18.6	45.6
Highest	6,589	29.3	37.2	72.6
**Place of residence**				
Urban	6,918	25.9	33.0	63.9
Rural	13,182	22.9	11.4	30.3
**Region**				
North-central	3,617	31.7	15.3	52.2
North-east	4,241	19.5	3.9	25.3
North-west	6,136	12.5	3.7	15.4
South-east	2,083	32.5	39.3	82.0
South-south	1,755	26.9	34.7	52.1
South-west	2,268	32.8	62.3	82.9
**Decision on respondent's healthcare**				
Husband/partner alone and other	8,066	20.5	11.2	30.0
Respondent and partner	5,481	28.8	28.4	60.0
Respondent alone	1,474	26.5	36.3	56.5
**Decision on large household purchase**				
Husband/partner alone and other	8,676	20.5	13.0	31.6
Respondent and partner	5,546	29.1	28.0	58.5
Respondent alone	766	28.9	32.3	62.6
**Decision on how to spend respondent's Earnings**				
Husband/partner alone and other	784	27.8	14.5	39.8
Respondent and partner	2,143	31.0	33.2	67.9
Respondent alone	6,727	22.4	22.5	43.3
**Decision on visits to family or relatives**				
Husband/partner alone and other	5,847	19.6	11.3	28.9
Respondent and partner	7,182	27.7	24.4	53.0
Respondent alone	1,992	24.8	26.1	48.2

#### Timing of first ANC visit

The results indicated that a high proportion of first ANC visits before 12 weeks of pregnancy was reported among women aged 15–24 years and 35 years or older; who had secondary/tertiary education; those who were employed; found in the highest wealth quintile households; resided in urban areas; and found in the South-west region. Considering women decision-making measures, initiating first ANC visits before 12 weeks of pregnancy was high among women who made joint decisions with partners on their healthcare (28.8%), large household purchases (29.1%), how to spend their earnings (31.0%) and visits to family or relatives (27.7%).

#### Number of ANC visits

The results on the number of ANC visits showed similar results recorded for socio-economic and demographic characteristics of the women and the timing of first ANC visits. The proportion of women that had at least eight ANC visits was high among those who made independent decisions on their healthcare (36.3%), large household purchases (32.3%) and visits to family or relatives (26.1%). On the other hand, the proportion was high among women who made joint decisions on how their earnings are spent (33.2%).

#### Facility delivery

Concerning socio-economic and demographic characteristics of the women and delivery in a health facility, similar results observed on the timing of the first ANC visit and the number of ANC visits was recorded. The results further showed that the proportion of women who delivered in a health facility was high among those who made joint decisions with partners on their healthcare (60.0%), how to spend their earnings (67.9%) and their visits to family or relatives (53.0%). However, the proportion was high among women who made independent decisions on large household purchases (62.6%).

### Unadjusted logistic regression analyses of the relationship between explanatory variables and antenatal care utilisation and institutional delivery services

Table 3 presented the unadjusted logistic regression analyses was adopted to measure the bivariate relationship between the outcome variables and each of the main explanatory variables, as well as co-variables.

**Table 3:** Unadjusted logistic regression model of the association between the explanatory variables and antenatal care visits and institutional delivery, 2018 Nigeria DHS

Variable/category	Timing of first ANC visit	Number of ANC visit	Facility delivery
	OR(95% CI)	OR(95% CI)	OR(95% CI)
**Decision on respondent's healthcare**			
Husband/partner alone and other (Ref.)	1.00	1.00	1.00
Respondent and partner	1.57(1.45–1.70)***	3.16(2.92–3.42)***	3.49(3.28–3.72)***
Respondent alone	1.39(1.22–1.58))***	4.53(4.05–5.08)***	3.03(2.73–3.35)***
**Decision on large household purchase**			
Husband/partner alone and other (Ref.)	1.00	1.00	1.00
Respondent and partner	1.59(1.47–1.72)***	2.60(2.41–2.80)***	3.06(2.88–3.26)***
Respondent alone	1.57(1.34–1.85)***	3.19(2.75–3.69)***	3.63(3.16–4.16)***
**Decision on how to spend respondent's Earnings**			
Husband/partner alone and other (Ref.)	1.00	1.00	1.00
Respondent and partner	1.17(0.97–1.40)	2.92(2.42–3.53)***	3.20(2.76–3.71)***
Respondent alone	0.75(0.64–0.89)**	1.71(1.43–2.03)***	1.15(1.02–1.31)*
**Decision on visits to family or relatives**			
Husband/partner alone and other (Ref.)	1.00	1.00	1.00
Respondent and partner	1.58(1.45–1.71)***	2.53(2.33–2.74)***	2.77(2.60–2.95)***
Respondent alone	1.36(1.20–1.53)***	2.76(2.47–3.09)***	2.28(2.08–2.50)***
**Age**			
15 – 24(Ref.)	1.00	1.00	1.00
25 – 34	1.05(0.95–1.15)	1.44(1.34–1.54)***	1.55(1.44–1.66)***
35 +	1.00(0.90–1.11)	1.39(1.29–1.50)***	1.52(1.44–1.66)***
**Educational attainment**			
No education (Ref.)	1.00	1.00	1.00
Primary	1.60(1.42–1.80)***	3.28(3.02–3.57)***	4.46(4.08–4.89)***
Secondary or higher	2.31(2.11–2.53)***	7.89(7.35–8.47)***	14.42(13.42–15.55)***
**Employment status**			
Not working (Ref.)	1.00	1.00	1.00
currently working	1.36(1.25–1.48)***	2.05(1.93–2.17)***	2.17(2.04–2.31)***
**Wealth quintile**			
Lowest (Ref.)	1.00	1.00	1.00
Middle	1.24(1.11–1.37)***	2.81(2.60–3.03)***	3.68(3.40–3.99)***
Highest	1.73(1.59–1.89)***	7.09(6.58–7.64)***	11.66(10.82–12.57)***
**Place of residence**			
Urban (Ref.)	1.00	1.00	1.00
Rural	0.85(0.79–0.91)***	0.30(0.28–0.31)***	0.25(0.23–0.26)***
**Region**			
North-central (Ref.)	1.00	1.00	1.00
North-east	0.52(0.46–0.59)***	0.58(0.53–0.64)***	0.31(0.28–0.34)***
North-west	0.31(0.27–0.35)***	0.51(0.47–0.55)***	0.17(0.15–0.18)***
South-east	1.04(0.91–1.17)	4.33(3.77–4.97)***	4.18(3.67–4.75)***
South-south	0.79(0.68–0.91)***	1.58(1.40–1.78)***	1.00(0.89–1.12)
South-west	1.05(0.93–1.18)	5.76(4.98–6.67)***	4.43(3.90–5.03)***

Note: *p < 0.05; **p < 0.01; ***p < 0.001; *Ref*. = reference category

#### Timing of first ANC visit

The results of the unadjusted logistic regression analyses are presented in Table 3 indicated that all the women’s decision-making variables were significant with the timing of the first ANC visit. The results showed that the likelihood of having first ANC visit before 12 weeks of pregnancy significantly increased among women who made joint decisions with partners and independent decisions on their healthcare, large household purchases and visits to family or relatives, relative to those in the reference category. On the other hand, the likelihood of having first ANC visits before 12 weeks of pregnancy significantly reduced among women who made independent decisions on how their earnings are spent (OR: 0.75; CI: 0.64–0.89). For socio-economic and demographic variables, except for age, all the other variables were significantly related to the timing of the first ANC visit.

#### Number of ANC visits

As observed in the timing of the first ANC visit, the results revealed that women whose husbands/partners made independent decisions on their healthcare (OR: 0.37, CI: 0.34–0.42), large household purchases (OR: 0.38, CI: 0.33–0.44), how to spent their earnings (OR: 0.72, CI: 0.63–0.81) and visits to family or relatives (OR: 0.53, CI: 0.49–0.58) significantly reduced the odds of having at least four ANC visits. Besides, the co-variables that significantly influenced the likelihood of having at least four ANC visits included age, maternal education, employment status, household wealth quintile, place of residence, as well as region of residence.

#### Facility delivery

 In Table 3, the results showed that women who made independent decisions and joint decisions with partners on their healthcare, large household purchases, how their earnings are spent and visits to family or relatives were significantly more likely to deliver in health facilities than their counterparts in the reference categories. All the socio-economic and demographic variables were significantly associated with delivery in a health facility.

### Adjusted logistic regression analyses

The adjusted logistic regression analyses results presented in Table 4 showed some variations in the timing of the first ANC visit, the number of ANC visits and facility delivery by women’s decision-making measures and selected socio-economic and demographic variables.

**Table 4:** Adjusted logistic regression analyses of women’s decision-making measures, selected co-variables and outcome variables, 2018 Nigeria DHS

	Timing of first ANC visit		Number of ANC visit		Facility delivery	
	Model 1	Model 2	Model 1	Model 2	Model 1	Model 2
Variable/category	aOR(95% CI)	aOR(95% CI)	aOR(95% CI)	aOR(95% CI)	aOR(95% CI)	aOR(95% CI)
**Decision on respondent's healthcare**						
Husband/partner alone and other (Ref.)	1.00	1.00	1.00	1.00	1.00	1.00
Respondent and partner	1.12(0.96–1.30)	0.93(0.79–1.09)	1.84(1.59–2.13)***	0.90(0.76–1.08)	1.84(1.63–2.09)***	1.09(0.94–1.28)
Respondent alone	1.21(1.10–1.46)*	0.94(0.77–1.14)	2.94(2.49–3.47)***	1.24(1.02–1.51)*	2.24(1.93–2.60)***	1.05(0.87–1.27)
**Decision on large household purchase**						
Husband/partner alone and other (Ref.)	1.00	1.00	1.00	1.00	1.00	1.00
Respondent and partner	1.16(1.00–1.36)	1.01(0.87–1.19)	1.53(1.32–1.76)***	1.02(0.86–1.21)	1.48(1.31–1.68)***	0.92(0.79–1.07)
Respondent alone	1.41(1.14–1.74)**	1.56(0.93–1.44)	1.63(1.34–1.98)***	0.87(0.69–1.08)	2.10(1.74–2.53)***	1.09(0.87–1.37)
**Decision on how to spend respondent's** **Earnings**						
Husband/partner alone and other (Ref.)	1.00	1.00	1.00	1.00	1.00	1.00
Respondent and partner	0.88(0.72–1.07)	0.85(0.70–1.03)	1.57(1.28–1.92)***	1.61(1.29–2.01)***	1.54(1.31–1.82)***	1.09(0.96–1.24)
Respondent alone	0.66(0.56–0.78)***	0.75(0.63–0.89)**	1.27(1.06–1.52)*	2.02(1.64–2.48)***	0.85(0.74–0.97)*	0.89(0.76–1.06)
**Decision on visits to family or relatives**						
Husband/partner alone and other (Ref.)	1.00	1.00	1.00	1.00	1.00	1.00
Respondent and partner	1.31(1.13–1.52)***	1.28(1.10–1.49)**	1.00(0.86–1.45)	0.88(0.74–1.04)	1.20(1.07–1.34)**	1.22(1.01–1.48)*
Respondent alone	1.18(0.99–1.41)	1.20(1.00–1.43)*	1.09(0.93–1.29)	1.02(0.85–1.25)	1.09(0.95–1.25)	1.12(0.95–1.32)
**Educational attainment**						
No education (Ref.)		1.00		1.00		1.00
Primary		1.08(0.92–1.28)		1.55(1.28–1.88)***		1.91(1.67–2.19)***
Secondary or higher		1.48(1.26–1.73)***		2.33(1.95–2.79)***		3.85(3.39–4.38)***
**Employment status**						
Not working (Ref.)		1.00		1.00		1.00
currently working		0.89(0.70–1.13)		0.99(0.74–1.32)		0.76(0.61–0.95)*
**Wealth quintile**						
Lowest (Ref.)		1.00		1.00		1.00
Middle		0.95(0.82–1.10)		1.60(1.35–1.88)***		1.85(1.63–2.09)***
Highest		1.10(0.95–1.28)		2.10(1.79–2.46)***		3.16(2.77–3.61)***
**Place of Residence**						
Urban (Ref.)		1.00		1.00		1.00
Rural		0.13(0.01–0.26)*		0.72(0.64–0.80)**		0.84(0.75–0.93)**
**Region**						
North-central (Ref.)		1.00		1.00		1.00
North-east		0.66(0.55–0.80)***		0.25(0.19–0.33)***		0.48(0.41–0.56)***
North-west		0.48(0.40–0.57)***		0.29(0.23–0.35)***		0.27(0.23–0.31)***
South-east		1.01(0.85–1.19)		2.14(1.81–2.54)***		1.73(1.44–2.07)***
South-south		0.75(0.63–0.91)**		1.98(1.66–2.36)***		0.41(0.35–0.48)***
South-west		1.12(0.96–1.31)		4.78(4.09–5.60)***		1.94(1.64–2.30)***

Note: *p < 0.05; **p < 0.01; ***p < 0.001; Ref. = reference category

#### Timing of first ANC visit

In Model 1, the results indicated that the likelihood of initiating the first ANC visit before 12 weeks of pregnancy significantly increased among women who made independent decisions on their healthcare (aOR: 1.21, CI: 1.10–1.46) and large household purchases (aOR: 1.41, CI: 1.14–1.76). The women who made joint decisions with partners on visits to family or relatives were significantly more likely to have their first ANC visits before 12 weeks of pregnancy (aOR: 1.31, CI: 1.31–1.52) than those in the reference category. Surprisingly, the likelihood of initiating first ANC visits before 12 weeks of pregnancy was significantly reduced among women who made independent decisions on how their earnings are spent (aOR: 0.66, CI: 0.56–0.78). Similar results of the likelihood of having first ANC visits before 12 weeks of pregnancy among women who made independent decisions on how their earnings are spent were recorded after adjusting with selected socio-economic and demographic variables in Model 2. Also, women who made joint decisions with partners and those who made independent decisions on their visits to family or relatives were significantly more likely to have first ANC visits before 12 weeks of pregnancy relative to those in the reference category. The results in Model 2 further showed some variations among the selected socio-economic and demographic variables. For instance, women who were rural residents were significantly less likely to have their first ANC visits before 12 weeks of pregnancy (aOR: 0.13, CI: 0.01–0.26) than their urban counterparts. There were some differences recorded across the geopolitical regions in terms of timing of first ANC visits among the women.

#### Number of ANC visits

In Model 1, the results on having at least eight ANC visits showed almost similar patterns of the influence of women’s decision-making measures. For instance, the likelihood of having at least eight ANC visits during pregnancy was significantly increased among women who made joint decisions with partners and independent decisions on their healthcare, large household purchases and how their earnings are spent. The results presented in Model 2 showed that the likelihood of having at least eight ANC visits was significantly reduced among women who made independent decisions on their healthcare (aOR: 1.24, CI: 1.02–1.51), those who made joint decisions joint decisions with partners (aOR: 1.61, CI: 1.29–2.01) and independent decisions (aOR: 2.02, CI: 1.64–2.48) on how their earnings are spent. The results further showed some differences regarding the influence of the selected socio-economic and demographic variables on having at least eight ANC visits among women during pregnancy.

#### Facility delivery

The adjusted logistic regression analysis results presented in Table 4, Model 1 showed that women who made joint decisions with partners and independent decisions on their healthcare and large household purchases were more likely to deliver in a health facility than those in the reference category. Similar results were observed among women who made joint decisions on how their earnings are spent (aOR: 1.54, CI: 1.31–1.82) and visits to family or relatives (aOR: 1.20, CI: 1.07–1.34). Surprisingly, the likelihood of delivering in a health facility was significantly reduced among women who made independent decisions on how their earnings are spent (aOR: 0.85, CI: 0.74–0.97) compared to those in the reference category. After adjusting for selected co-variables in Model 2, it was revealed that the likelihood of delivering in a health facility significantly increased among women who made joint decisions with partners on their visits to family or relatives (aOR: 1.22, CI: 1.01–1.48). The results further indicated that an increase in women's level of education and household wealth quintile increased the likelihood of delivering in a health facility. Unexpectedly, the results showed that the likelihood of delivering in a health facility significantly reduced among women who reported to be employed (aOR: 0.76, CI: 0.61–0.95). There were significant variations relating to delivery in a health facility among women by place of residence and region.

## Discussion

This study examined the influence of women’s decision-making autonomy on ANC utilisation and institutional delivery services among women in Nigeria using the 2018 NDHS survey. Using a nationally representative population sample of childbearing women who were currently married and living with partners, with a special focus on the indicators of maternal healthcare services utilisation during pregnancy and childbirth. This marks a departure in terms of its national coverage from most of the previous studies on maternal health services utilization in Nigeria.

Maternal healthcare services utilisation during pregnancy and childbirth is important to reduce preventable maternal and newborn mortality and morbidity through the management of pregnancy-related complications [39, 40]. Overall, the findings of this study revealed that while 75.6% of the women initiated first ANC visits before 12 weeks of pregnancy, 75.8% had less than eight ANC visits as against the revision of the WHO’s four-visit focused ANC (FANC) model recommending the minimum number of ANC visits from four to eight contacts throughout the pregnancy. The finding on the timing of the first ANC is in contrast to the observation of a previous study in Tanzania which demonstrated that timely attendance at the ANC in the first trimester of pregnancy was low [40]. The findings further revealed that over one-half of the women delivered their babies at home during their last pregnancies. This is an indication that initiating the first ANC visits by pregnant women within the first trimester of pregnancy, may not translate to having at least eight ANC visits and delivery in a health facility. Also, the findings on decision-making revealed that an overwhelming majority of the women enjoyed decision-making autonomy on how to spend their earnings, unlike on issues relating to their healthcare, large household purchases and visits to family or relatives.

The findings on the influence of women’s decision-making measures on ANC utilisation and institutional delivery services showed that women’s decision-making autonomy on their healthcare was significantly found to be a protective factor to having at least eight ANC visits during pregnancy. This corroborates a previous study in Ghana that observed women’s limited autonomy creates a serious barrier to access and use maternal healthcare services [41]. In addition, the findings showed that women, who made joint decisions and independent decisions on how their earnings are spent, increased the likelihood of having at least eight ANC visits. Based on the findings on joint decision-making, in line with previous research in Bangladesh, this study establishes the significance of male partners being involved in decision-making on issues relating to maternal healthcare services as [42]. The findings further explain previous studies’ observations in some countries that women’s decision-making autonomy and participation increase the utilisation of maternal health services [43, 44]. Plausibly, this could be attributed to the danger of men’s independent decision-making within the family which might make it impossible for women to have the opportunity to voice out their minds, hence influencing quality ANC utilisation and delivery in a health facility as shown in a study [45].

Surprisingly, in contrast with previous studies in Nigeria [17, 20] and other countries [43, 44, 46–48], this study revealed that women’s decision-making autonomy on how their earnings are spent was significantly found to be an inhibiting factor to initiating ANC visits early. This explains how women’s perceived benefits of behavioural intentions influence the performance of such behaviours [49]. Consequently, most women who had decision-making autonomy on how to spend their earnings might be constrained to postpone early initiation of ANC visits and reduce the number of ANC visits, as well as consider delivering at home. This plausibly might be an opportunity for them to financially take care of some urgent family issues, especially in the absence of their husbands/partners. In line with Dudgeon & Inhorn’s observation [19], this becomes inimical to policy interventions towards achieving the SDGs targets of quality and universal access to reproductive healthcare services and reducing maternal and child deaths. This is also important considering the negative impacts of the Coronavirus pandemic and socio-economic lockdown on accessing quality ANC utilisation and delivery in a health facility in Nigeria.

This study revealed that women with secondary or higher education were significantly more likely to initiate first ANC visits before 12 weeks of pregnancy. The findings further established a significantly direct influence of women’s educational attainment on having at least eight ANC visits and delivering in a health facility. These corroborate the findings of previous research in some sub-Saharan African countries including Nigeria that women with higher education were more likely to be empowered in terms of knowledge about the availability and benefits of maternal health services, as well as make independent decisions regarding their health [23, 50–52]. Also, women’s household wealth quintiles have significant linear relationships with having at least eight ANC visits and delivery in a health facility. No doubt, women’s household wealth quintile is closely related to their educational attainment and employment status. Educated women tend to be more employed and consequently earn more income which provides them with the opportunity to have quality ANC utilization and delivery in a health facility. There is the need to introduce and encourage more women education and empowerment programmes geared towards promoting access to quality ANC utilisation and delivery in a health facility in Nigeria.

With respect to the place of residence, the findings established that the likelihood of initiating first ANC visits before 12 weeks of pregnancy, having at least eight ANC visits and delivering in a health facility was significantly reduced among women who were rural residents. The findings could be explained by the urban–rural disparity regarding peculiar characteristics such as quality educational attainment, employment status and exposure to mass media of residents, as well as the location of health centres as shown in previous studies conducted in South Africa and Guinea [53, 54]. The findings further revealed some regional variations on initiation of first ANC visits before 12 weeks of pregnancy, having at least eight ANC visits and delivery in a health facility. This validates previous observations in Nigeria that residing in a particular geographic region influenced women’s ANC utilisation and institutional delivery services [22, 23, 26]. There is the need to encourage quality ANC utilisation and delivery in the health facility among pregnant women across the six geopolitical regions, especially disadvantaged regions through women empowerment programmes and region-wide sensitisations on maternal healthcare utilisation. Also, there should be more strategies towards directly engaging men in maternal health issues through couple counselling to further encourage access to quality ANC utilisation and delivery in the health facility among women. This is imperative in a country like Nigeria where there is a strong adherence to patriarchal norms and cultural perceptions of men.

The outcomes of this study have some policy implications and interventions towards achieving the SDGs targets of quality and universal access to maternal healthcare services, as well as reducing maternal and child deaths, through women empowerment programmes and region-wide sensitisations on maternal healthcare utilisation. However, there is a need for more quantitative and qualitative studies based on the disaggregation of the data between the geopolitical zones, to explore contextual and socio-cultural factors influencing ANC utilisation and institutional delivery services among childbearing women in Nigeria.

### Strengths and limitations of the study

The main strengths of this study are the focus on the up-to-date recommendation for ANC visits and the use of population-based data generated from the most recent Demographic and Health Survey, which is useful and ideal for future policy formulations. Also, the study used a national representative large sample and adopted rigorous analytical procedures with weighted proportions.

This study has its limitations. The use of cross-sectional DHS data meant cause-effect relationship could not be determined, and the explanatory variables were only temporal factors associated with quality ANC utilisation and institutional delivery services. There is the likelihood of reporting bias/discordance regarding decision-making autonomy within the family as the participants were only women and for the fact that it is to a large extent a subjective phenomenon. Also, the differences that exist between geopolitical regions in terms of some of the main outcome variables and explanatory variables could account for the results showing that despite a large number of women initiating antenatal care early, far fewer numbers delivered in a health facility. Despite these limitations, the findings are important for more strategic policies and programmes, especially for disadvantaged women concerning their decision-making powers influencing ANC utilisation and institutional delivery services which affect maternal and child health outcomes in Nigeria.

## Conclusion

This study revealed that despite a large number of women initiating ANC before 12 weeks of pregnancy, far less numbers had at least eight ANC visits and delivered their babies in health facilities. It was further established that women’s decision-making autonomy on their healthcare and how their earnings are spent was significantly found to be a protective factor to having at least eight ANC visits during pregnancy. Surprisingly, women’s decision-making autonomy on how their earnings are spent was significantly found to be an inhibiting factor to initiating ANC early. There should be more pragmatic efforts and programmes through a community and region-based campaign involving men to optimise maternal healthcare services utilisation, especially among the disadvantaged women through enlightenment and empowerment programmes. This becomes important towards improving maternal and child health outcomes in a patriarchal society like Nigeria where most women experience unequal power in their intimate relationships with opposite-sex partners.

## Data Availability

The NDHS 2018 individual recode dataset was used for this study and is freely available from the DHS Program archive at https://www.dhsprogram.com/data/dataset.

## Abbreviations

ANC: Antenatal care
aOR: Adjusted Odds Ratio
WHO: World Health Organisation
FANC: Focused antenatal care
NDHS: Nigeria Demographic and Health Survey
NPC: National Population Commission
NPHC: National Population and Housing Census
EA: Enumeration Area
LGA: Local Government Areas
OR: Odds Ratio
DHS: Demographic and Health Survey
CIs: Confidence intervals
RC: Reference category
SDGs: Sustainable Development Goals
COVID-19: Coronavirus
